# Novel aminopyridazine derivative of minaprine modified by radiolysis presents potent anti-inflammatory effects in LPS-stimulated RAW 264.7 and DH82 macrophage cells

**DOI:** 10.1038/s41598-023-37812-8

**Published:** 2023-07-05

**Authors:** Gyeong Han Jeong, Hanui Lee, So-Yeun Woo, Hong-Ki Lee, Byung Yeoup Chung, Hyoung-Woo Bai

**Affiliations:** 1 Research Division for Biotechnology, Advanced Radiation Technology Institute (ARTI), Korea Atomic Energy Research Institute (KAERI), Jeongeup, 56212 Republic of Korea; 2 Center for Companion Animal New Drug Development, Korea Institute of Toxicology (KIT), Jeongeup, 56212 Republic of Korea; 3 Radiation Biotechnology and Applied Radioisotope Science, University of Science and Technology (UST), Daejeon, 34113 Republic of Korea

**Keywords:** Biological techniques, Biotechnology, Drug discovery, Structural biology

## Abstract

Radiation molecularly transforms naturally occurring products by inducing the methoxylation, hydroxylation, and alkylation of parent compounds, thereby affecting the anti-inflammatory capacities of those compounds. Minaprine (**1**) modified by ionizing radiation generated the novel hydroxymethylation hydropyridazine (**2**), and its chemical structure was determined based on NMR and HRESIMS spectra. Compared to the original minaprine, the novel generated product showed a highly enhanced anti-inflammatory capacity inhibited nitric oxide (NO) and prostaglandin E_2_ (PGE_2_) production in lipopolysaccharide (LPS)-stimulated RAW 264.7 and DH82 macrophage cells. In addition, minaprinol (**2**) effectively inhibited cyclooxygenase-2 (COX-2) and inducible NO synthase (iNOS) at the protein level and pro-inflammatory cytokine (tumor necrosis factor-α (TNF-α), interleukin-6 (IL-6), and IL-10) production in macrophages.

## Introduction

Inflammation occur in response to injury and involves localized accumulations of body fluids, plasma proteins, and white blood cells associated with immune system action or physical injury and infection^1,2^. Inflammatory responses lead to the excessive production of inflammatory mediators such as pro-inflammatory nitric oxide (NO), prostaglandin E_2_ (PGE_2_), tumor necrosis factor-α (TNF-α), and interleukin-6 (IL-6), or the inhibition of secretions by anti-inflammatory cytokines such as IL-10 and so on^3–5^. The formation of inflammatory mediators plays a major role in mediating inflammation based on their ability to convert arachidonic acid into leukotriene, thromboxane, and prostaglandins through the reaction of cyclooxygenase-2 (COX-2)^6^. In addition, NO production is regulated by inducible NO synthase (iNOS)^7^. The excessive inflammation can lead to a variety of chronic inflammation-based diseases, such as diabetes, cancer, arthritis, and cardiovascular disease^8^. These inflammatory diseases are fatal for humans as well as companion animals. Although research on drugs for inflammatory diseases in humans has been conducted, the development of similar prescription drugs for companion animals is limited. As the public adoption of companion animals has increased worldwide amidst the COVID-19 pandemic^9^, the related industries are also growing rapidly; therefore, additional prescription drugs must be developed for companion animals.

Gamma irradiation has received wide attention due to its potential to decrease the toxicity of alkaloids, steroids, and flavonoids and increase the biological capacities of these compounds^10^. Radiation transformation technology (RTT) for the development of candidate drugs has advantages of being simple, rapid, low cost, and environmentally friendly compared to organic solvent-based chemical synthesis^11^. Recently, the anti-depressant drug nomifensine (Merital®) was reported to effectively improve the anti-cancer properties in breast cancer cells after exposed to ionizing radiation^12^. Radiation induces the molecular transformation of natural products by inducing the methoxylation, hydroxylation, and alkylation of mother compounds^13,14^. However, studies on the structural modification of alkaloid-based drugs using γ-irradiation technology are still very limited. Thus, the exact chemical structure and anti-inflammatory potential of radiolytic molecules in aminopyridazine derivatives must be evaluated and better understood to promote their use in the treatment of inflammatory diseases.

The monoamine oxidase inhibitor, minaprine (Brantur®) is used as an anti-depressant and shows inhibitory effects on acetylcholinesterase in vivo; however, it was withdrawn from the anti-depressant drug market in 1996 because it causes convulsions^14,15^. Recently, a new class of aminopyridazine derivatives exhibiting various biological activities, such as anti-cancer, anti-diabetic, and cardiovascular activities, have been developed, and their value as functional materials has been demonstrated^16^. As part of our continuing search for novel drugs for humans and companion animals, we herein report the radiolytic modification of minaprine into a novel hydroxymethylated hydropyridazine derivative (**2**) that exhibits significantly enhanced anti-inflammatory properties compared to the original minaprine.

## Result and discussion

### Determination and isolation of newly generated product

**Figure 1:**
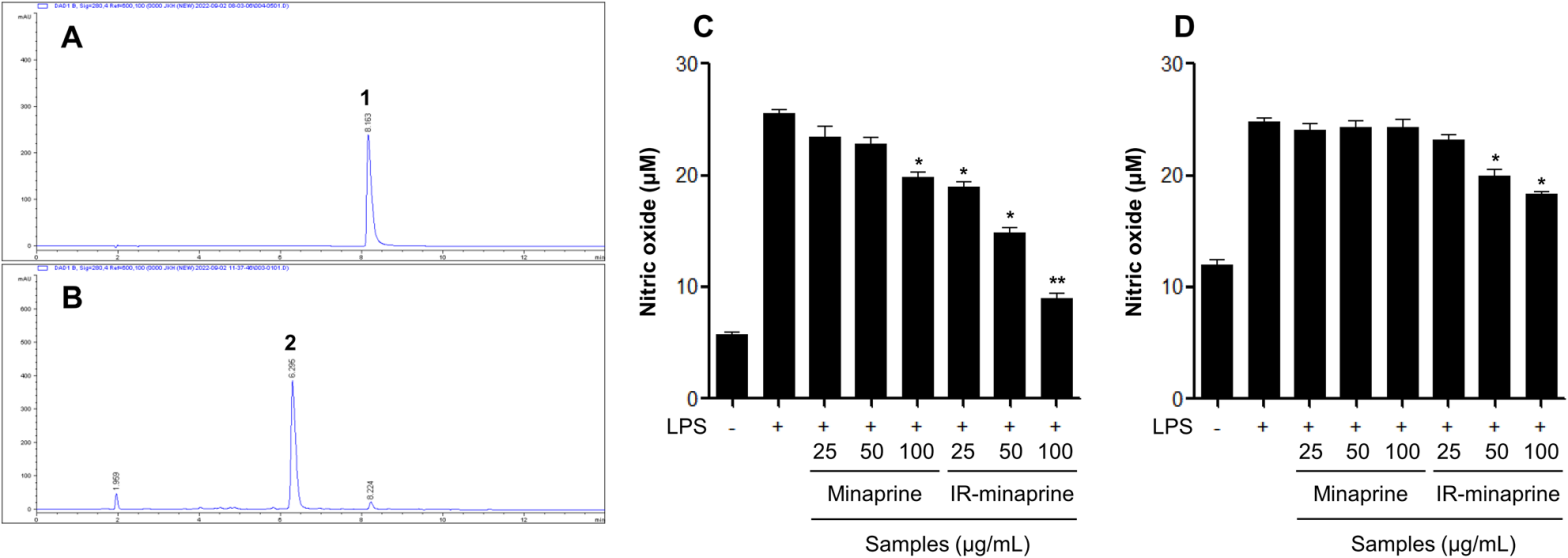
Effects of radiolytic transformation of minaprine. (**A**, **B**) HPLC chromatograms of (**A**) pure minaprine and (**B**) irradiated reactant at 30 kGy. (**C**, **D**): NO production of minaprine and irradiated minaprine (IR-minaprine) in (**C**) RAW 264.7 and (**D**) DH82 macrophage cells was analyzed by a Griess reagent assay. The results are expressed as mean ± SD (*n* = 3). *p < 0.05; **p < 0.01; *vs*. LPS-treated group. **1**: minaprine, **2**: minaprinol.

**Figure 2:**
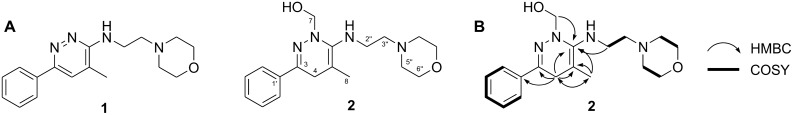
Structures of newly degraded product **2** from minaprine (**A**) and key HMBC and COSY correlations of **2** (**B**).

Ionizing radiation was applied as previously described. A sample solution containing pure minaprine (400 mg) in methanol (400 mL) was directly irradiated with gamma-rays, and the transformation pattern was analyzed using a reverse-phases HPLC apparatus. After irradiation with a dose of 30 kGy, minaprine (**1**) was nearly reduced and the newly generated peak at a *t*_R_ 6.2 min corresponded to a conversion rate of 95% (Fig. 1A,B ). The reactants irradiated at a dose of 30 kGy showed enhanced inhibitory effects on NO production in lipopolysaccharide (LPS)-stimulated RAW 264.7 and DH82 macrophages compared with pure minaprine (Fig. 1C,D). Repeated column chromatographic separation of the 30 kGy-irradiated reactants led to the isolation and purification of the novel hydropyridzine derivative **2**. The new hydroxyalkylated product **2** was found to contain rare functional groups with a nitrogen moiety at the aminopyridazine of minaprine (Fig. 2A).

Compound **2** was purified as a yellowish oil, and the absorption maxima at 219 and 284 nm in the UV spectrum suggested the presence of a hydropyridazine skeleton^17^. The HRESIMS revealed a pseudomoelcular ion peak at *m/z* 331.2126 [M + H]^+^ corresponding to the molecular formula C_18_H_27_N_4_O_2_. The ^1^H NMR spectrum (600 MHz) of **2** in CD_3_OD showed one benzene ring resonance signals at *δ*_H_ 7.90 (2H, dd, *J* = 8.4, 1.8 Hz, H-2′, 6′), 7.51 (1H, dd, *J* = 8.4, 1.8 Hz, H-4′), and 7.49 (2H, t, *J* = 8.4 Hz, H-3′, 5′), 6 methylene proton signals at *δ*_H_ 3.73 (4H, t, *J* = 4.2 Hz, H-6″, 7″), 3.61 (2H, t, *J* = 5.4 Hz, H-2″), 2.77 (2H, t, *J* = 5.4 Hz, H-3″), and 2.60 (4H, br s, H-5″, 8″), which suggested the presence of the one benzene and alkylmorpholine moieties^18^. One singlet methyl group at *δ*_H_ 1.36 (3H, s, H-8) and methylene protons at *δ*_H_ 2.94 (1H, d, *J* = 17.4 Hz, H-4a) and 2.88 (1H, d, *J* = 17.4 Hz, H-4b) were observed. In addition, the 1D NMR and HSQC spectra revealed the presence of a hydroxymethyl substituent at *δ*_H_ 3.80 (1H, d, *J* = 11.4 Hz, H-7a) and 3.71 (1H, d, *J* = 11.4 Hz, H-7b) (Table 1).

**Table 1:** ^1^H and ^13^C NMR spectroscopic data for new compound **2** (*δ* in ppm, *J* in Hz) in CD_3_OD.

Position	*δ*_H_, (*J* in Hz)	*δ*_C_, type	Position	*δ*_H_, (*J* in Hz)	*δ*_C_, type
3	–	157.8, C	3′	7.49 (t, 1.8)	127.5, CH
4a	2.94 (d, 17.4)	30.8, CH_2_	4′	7.51 (dd, 8.4, 1.8)	132.3, CH
4b	2.88 (d, 17.4)		5′	7.49 (t, 1.8)	127.5, CH
5	–	124.1, C	6′	7.90 (dd, 8.4, 1.8)	129.9, CH
6	–	164.9, C	2″	3.61 (t, 5.4)	41.6, CH_2_
7a	3.80 (d, 11.4)	67.1, CH_2_	3″	2.77 (t, 5.4)	56.6, CH_2_
7b	3.71 (d, 11.4)		5″	2.60 (br s)	54.1, CH_2_
8	1.36 (s)	19.9, CH_3_	6″	3.73 (t, 4.2)	67.9, CH_2_
1′	–	136.1, C	7″	3.73 (t, 4.2)	67.9, CH_2_
2′	7.90 (dd, 8.4, 1.8)	129.9, CH	8″	2.60 (br s)	54.1, CH_2_

Eighteen carbon signals observed in the ^13^C NMR and HSQC spectra could be classified as 9 *sp*^2^ carbons (assigned to one aromatic ring and three olefins), 8 methylene carbons at *δ*_C_ 67.9 (C-6″, 7″), 67.1 (C-7), 56.6 (C-3″), 54.1 (C-5″, 8″), 41.6 (C-2″), and 30.8 (C-4), and one methyl signal at *δ*_C_ 19.9 (C-8) (Table 1). The above data are indicative of an unusual hydropyridazine with hydroxymethyl moieties (Fig. 2A). The structure of **2** was further confirmed by HMBC. The HMBC relationships from H-4 to C-3, -5, and -6, from H-8 to C-4 and -6, from H-1′ to C-3, and from H-2″ to C-6, confirmed that **2** has an aminopyridazine backbone. The cross peaks at H-4, -8, and -2″ to C-6 confirmed that **2** is a 3-benznyl-8-methyl-6-aminopyridazine with morpholine located at C-6. The hydroxymethyl functionalities were located at the nitrogen position of the hydrated aminopyridazine unit as supported by the key HMBC correlation between H-7 and C-6 (Fig. 2B). Thus, the structure of the unusual hydroaminopyridazine **2** was fully assigned as minaprinol, which is a novel transformation product, as shown in Fig. 2A.

Radiolysis of methanolic conditions in the presence of an atmosphere produces various free radicals, such as hydroxymethyl (^·^CH_2_OH), hydroxyl (^•^OH), methoxy (CH_3_O^·^), and peroxyl (HOO^·^), which are subsequently green-synthesis of new drug candidates^19,20^. Our results suggest that the hydroxymethyl (^·^CH_2_OH) radical may also be involved in the modification of compound **2** when minaprine is exposed to ionizing radiation in methanolic solution. This is the first example of the novel radiolytic hydroxymethylation of the amionpyridazine drugs.

### Inhibitory effects of anti-inflammatory in LPS-stimulated macrophage cells

**Figure 3:**
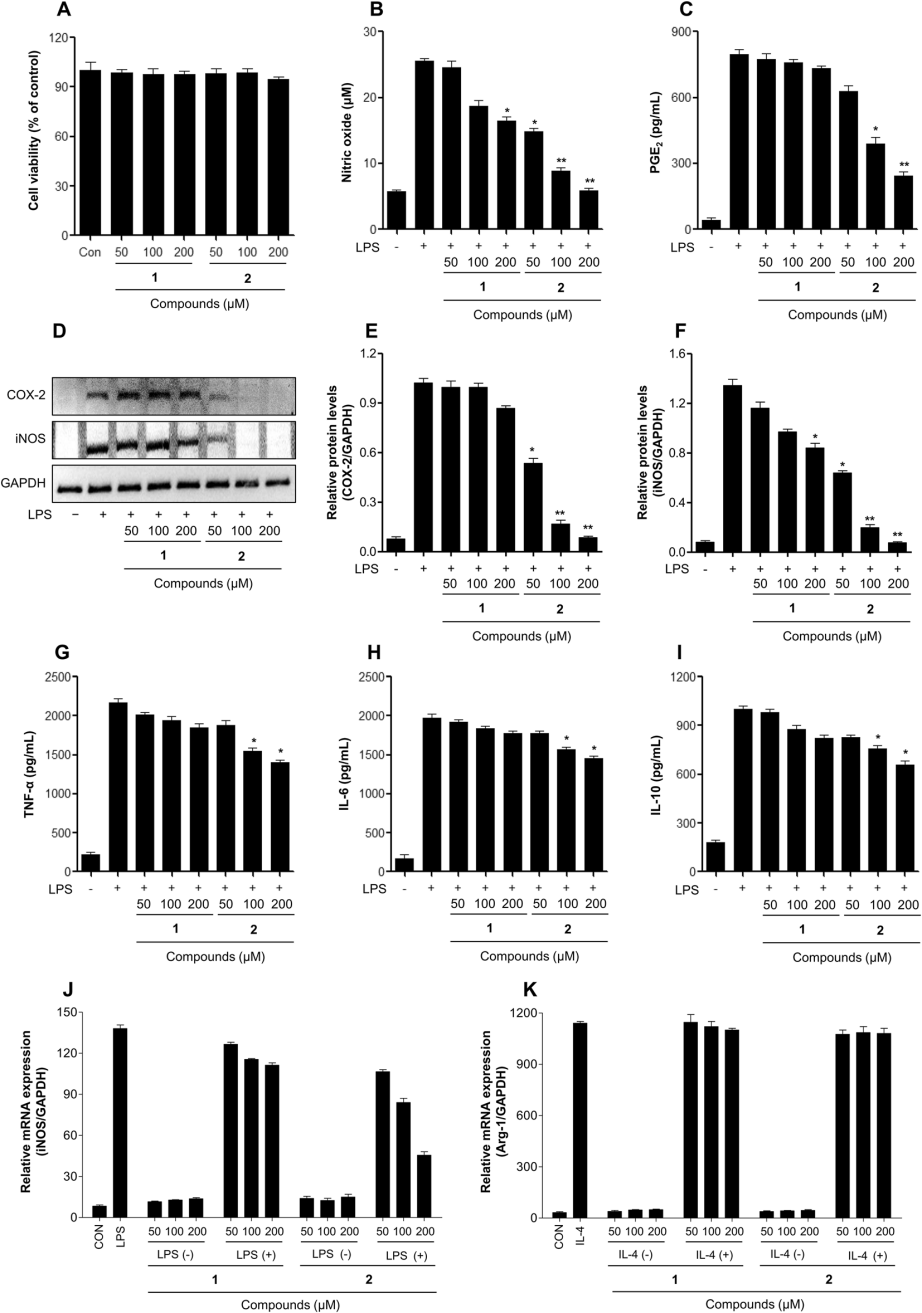
Effects of the aminopyridazine product on anti-inflammatory activities in LPS-stimulated RAW 264.7 macrophage cells. (**A**) Cell viability detection by MTT assay in RAW 264.7 cells after treatment with samples for 24 h. The cells were treated with samples for 2 h, followed by treatment with the LPS (0.1 μg/mL) for 24 h. (**B**) The nitrite content of culture media was analyzed by a Griess reagent assay. (**C**) The PGE_2_ production content of culture media was analyzed by ELISA. (**D**, **E**, **F**) Expression of COX-2 (**E**) and iNOS (**F**) induced by samples treatment of both RAW 264.7 macrophage cells for 24 h. (**G**, **H**, **I**) Cytokine productions (**G**: TNF-α, **H**: IL-6, **I**: IL-10) of culture media was measured by ELISA. (**J**, **K**) The iNOS (**J**) and Arg-1 (**K**) mRNA expression was determined by qPCR analysis. The relative expression of each gene was quantified using the expression level of GAPDH mRNA. The results are expressed as mean ± SD (*n* = 3). *p < 0.05; **p < 0.01; *vs*. LPS-treated group. **1**: minaprine, **2**: minaprinol.

**Figure 4:**
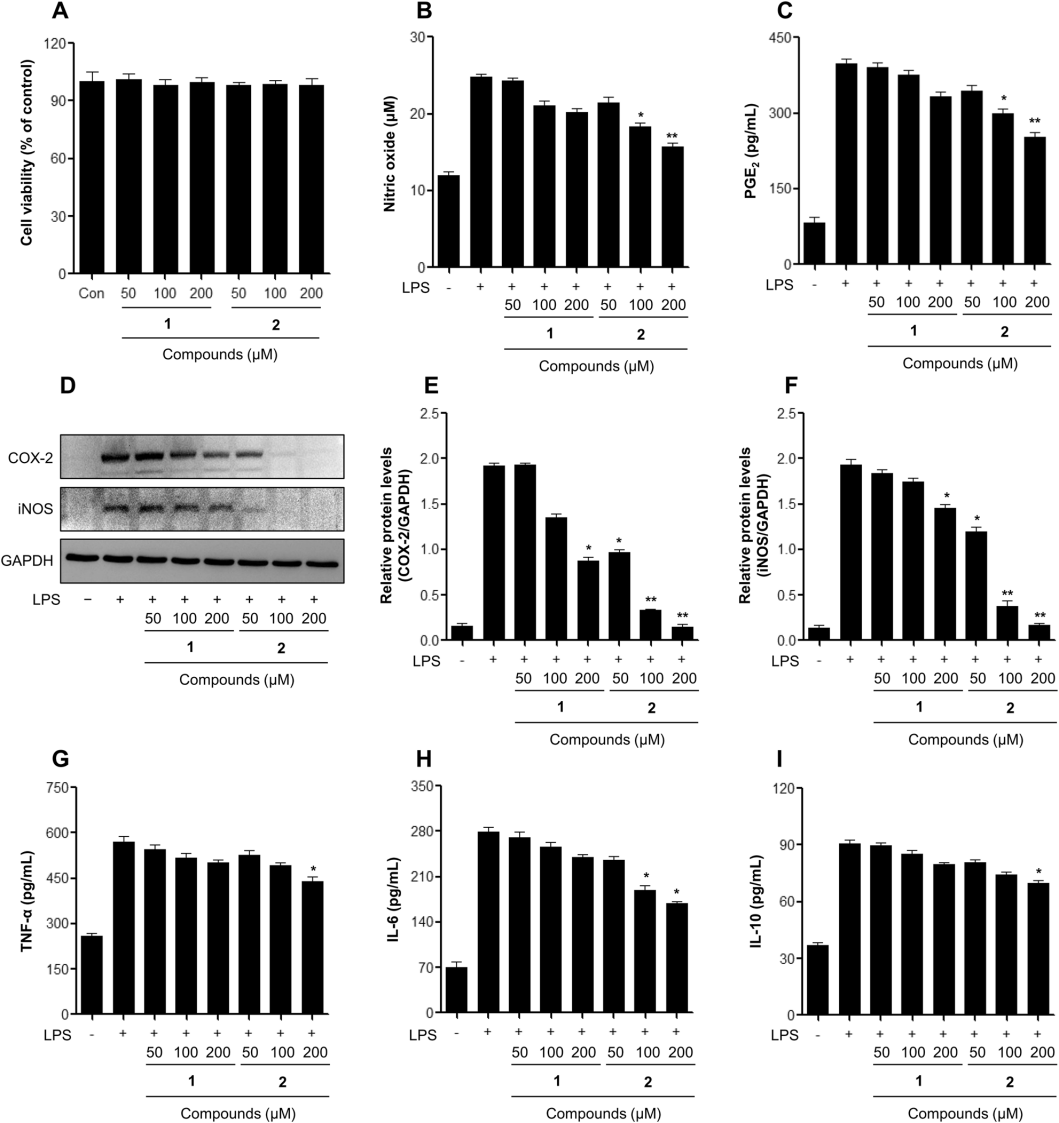
Effects of the aminopyridazine product on anti-inflammatory activities in LPS-stimulated DH82 macrophage cells. (**A**) Cell viability detection by MTT assay in DH82 cells after treatment with samples for 24 h. The cells were treated with samples for 2 h, followed by treatment with the LPS (1 μg/mL) for 24 h. (**B**) The nitrite content of culture media was analyzed by a Griess reagent assay. (**C**) The PGE_2_ production content of culture media was analyzed by ELISA. (**D**, **E**, **F**) Expression of COX-2 (**E**) and iNOS (**F**) induced by samples treatment of both DH82 macrophage cells for 24 h. (**G**, **H**, **I**) Cytokine productions (**G**: TNF-α, **H**: IL-6, **I**: IL-10) of culture media was measured by ELISA. The results are expressed as mean ± SD (*n* = 3). *p < 0.05; **p < 0.01; *vs*. LPS-treated group. **1**: minaprine, **2**: minaprinol.

The planner structure characterized for compound **2** was evaluated to determine its anti-inflammatory activity in both RAW 264.7 and DH82 macrophage cells. Treatment with compounds **1** and **2** did not affect the viability of RAW 264.7 and DH82 cells at 5% or less (up to 200 μM) (Figs. 3A and 4A). We evaluated the anti-inflammatory effects of the novel hydropyridazine derivative minaprinol (**2**) by detecting the production of pro-inflammatory mediators including NO and PGE_2_ in LPS-stimulated RAW 264.7 and DH82 macrophages (Figs. 3 and 4).

Minaprinol (**2**) possesses hydroxymethyl functionality, and at concentrations of 200 μM, it showed potent enhanced inhibitory activities against NO and PGE_2_ production in LPS-stimulated RAW 264.7 cells, with reductions of approximately 10.6 μM and 490.4 pg/mL relative to the parent minaprine, respectively (Fig. 3B,C). In DH82 cells, significant suppression of NO and PGE_2_ was observed (Fig. 4B,C). During inflammation, the enzymes COX-2 and iNOS promote the secretion of pro-inflammatory mediators^21^. Thus, **2** was investigated by western blot analysis to determine whether it inhibited COX-2 and iNOS in LPS-induced macrophage cells. As shown in Figs. 3D and 4D, compound **2** suppressed LPS-induced COX-2 and iNOS protein expression in a dose-dependent manner, resulting in the inhibition of pro-inflammatory mediator production, such as NO and PGE_2_.

Changes in the secretion of pro-inflammatory cytokines, including TNF-α and IL-6, and anti-inflammatory cytokines, such as IL-10, by LPS-stimulated macrophages were determined. Figure 3G‒**I** displays the inhibitory effects of the newly generated product **2** from irradiated minaprine on the secretion of inflammatory cytokines TNF-α, IL-6, and IL-10 by LPS-stimulated RAW 264.7 cells (Fig. 3). Minaprinol (**2**) also showed increased inhibition of pro- and anti-inflammatory cytokine production compared to minaprine (**1**) in DH82 cells from canine (Fig. 4G–I). The newly generated candidate **2** did not affect the mRNA expression levels of iNOS and Arg-1 in RAW 264.7 macrophage, which suggested did not involve in macrophage polarization (Fig. 3J,K). Interestingly, the hydroxymethylation of aminopyridazine induced by ionizing radiation improved the anti-inflammatory properties of LPS-stimulated macrophages. In a recent study, chrysin and luteolin showed improved anti-inflammatory activities in vitro and in vivo induced by γ-irradiation, and the chemical structure of hydroxyalkyl-substituted compounds was identified^12,21^. In addition, radiolytic transformation of alkaloids and steroids showed potent enhanced anti-cancer effects in melanoma, breast, liver, and lung cancer^11,22–25^.

### Colour change and modification of anti-inflammatory agents from minaprine by radiolysis

**Figure 5:**
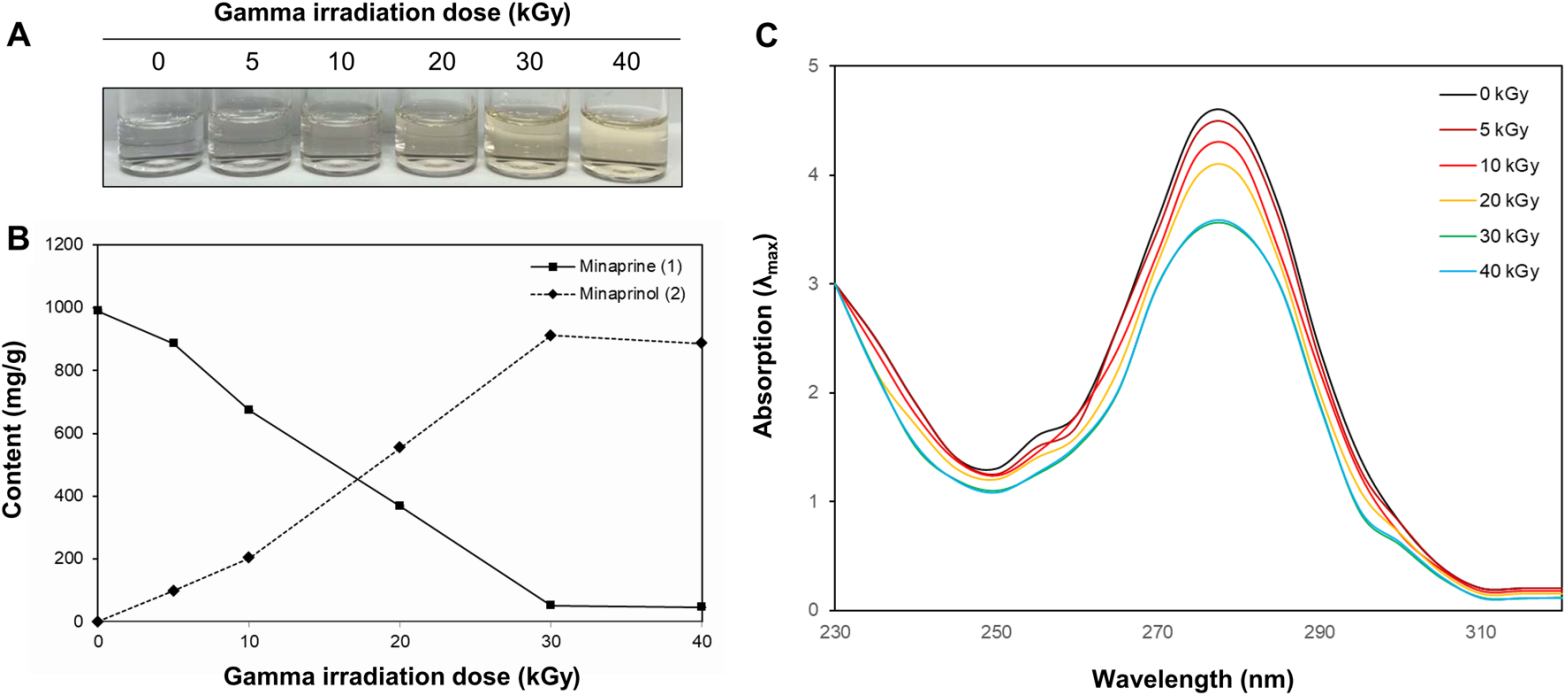
Change of physicochemical property of minaprine by gamma irradiation. (**A**) The colour changes in minaprine methanolic conditions following gamma irradiation was monitored visually. (**B**) Newly generated minaprinol (**2**) from minaprine (**1**) transformed by gamma irradiation were analyzed by HPLC at 280 nm. (**C**) Change of UV absorptions (λ_max_) of minaprine transformed by gamma irradiation dose.

The effects of ionizing irradiation on the colour changes and modification of new compound caused by hydroxymethylation of minaprine, which is most common type of hydroaminopyridazine, were examined. The white color of minaprine was gradually reduced in a dose-dependent manner and was effectively transformed at 30 kGy dose of gamma irradiation (Fig. 5A).

Contents of the isolated compound from the irradiated mianprine of 5, 10, 20, 30, and 40 kGy were quantified using the external standard method and the results are shown in Fig. 5B. Five concentrations points (*n* = 5) were used for the preparation of the calibration curve and the calibration curve of pure solutions of the standard compounds was completely linear (*R*^2^ > 0.999). The retention times of newly formed minaprinol (**2**, *t*_R_ 6.3 min) and minaprine (**1**, *t*_R_ 8.2 min) were detected for five irradiated reactants. Quantitative analysis revealed that the absolute contents of the most potent minaprinol (**2**) in the irradiated samples at 5, 10, 20, 30, and 40 kGy were 98.6 ± 0.6, 202.7 ± 1.5, 554.6 ± 1.6, 912.0 ± 2.0, and 886.0 ± 2.0 mg/g, respectively, which is in accordance with improvement of the anti-inflammatory capacity in macrophage cells of each sample. In contrast, reduction of minaprine (**1**) content was observed by ionizing irradiation.

The chemical characteristics of the modified minaprine were checked by UV spectrometry (Fig. 5C). The absorbance at 280 nm was attributed to the pyridazine bonds of minaprine (**1**). In contrast, 30 kGy-irradiated samples and decreased absorbance at 280 nm compared to that of control (0 kGy), which suggested greater transformation to dihydropyridazine backbone. These results suggest that the content of minaprinol (**2**) increased up to 30 kGy dose of ionizing radiation as the major product from minaprine. Our results indicate that hydroxymethylation induced by the application of ionizing radiation to minaprine may be beneficial in suppressing the generation of excessive inflammation in companion animal macrophage cells.

## Conclusion

In the present study, we first confirmed that minaprine is easily hydroxymethylated to the aminopyridazine compound **2**. Minaprinol (**2**) showed more potent anti-inflammatory activity toward RAW 264.7 and DH82 macrophage cells than the original minaprine. When mianprine was induced by ionizing radiation at 30 kGy, the conversion rate to minaprinol (**2**) was 95% or more, and it was efficiently produced (Fig. 1A and B). It would be interesting to extend the results to the companion animal drug industry for the development of novel anti-inflammatory therapeutics in dogs (as well as humans) based on hydroxymethylated minaprine derivatives. Overall, this study offers a unique approach to the production of novel aminopyridazines with greatly enhanced anti-inflammatory capacity.

## Methods

### Chemicals and instruments

Minaprine, acetonitrile, methanol, formic acid (HPLC grade), CD_3_OD, lipopolysaccharides (LPS), and Griess reagent were purchased from Sigma-Aldrich (St. Louis, MO, USA) or Merck (Darmstadt, Germany). All other reagents and chemicals purchased and used in this study were of analytical grade. Spectra of ^1^H and ^13^C nuclear magnetic resonance (NMR) were measured on a Avance NEO-600 instrument (Bruker, Karlsruhe, Germany) operated at 600 and 150 MHz, respectively. Chemical shifts are given in *δ* (ppm) values relative to those of the solvent CD_3_OD (*δ*_H_ 3.35; *δ*_C_ 49.0) on a tetramethylsilane (TMS) scale. The ESI mass spectra were measured by a Vanquish UPLS System (Thermo Fisher Scientific, MA, USA). YMC gel ODS AQ 120-50S (particle size 50 μm; YMC Co., Kyoto, Japan) was used for the column chromatogrphy, while a microplate reader (Infinite F200, Tecan Austria GmBH, Grodig, Austria) was used to measure the absorbance. A semi-prepratative high performance liquid chromatography (HPLC), Agilent HPLC 1200 system (Agilent Technologies, Palo Alto, CA, USA) equipped with a phtodiode array detector (PDA, 1200 Infinity series, Agilent Technologies) and a series of YMC-Pack ODS A-302 column (4.6 mm i.d. × 150 mm, particle size 5 μm; YMC Co., Kyoto, Japan) were used to purify the compound.

### Preparation of irradiated sample

Ionizing radiation was carried out at room temperature, using a cobalt-60 irradiator (Point source AELC, IR-79, MDS Nordion International Co. Ltd.) at the Advanced Radiation Technology Institute, Korea Atomic Energy Research Institute (Jeongup, Korea). The source strength was approximately 320 kCi with dose rate of 10 kGy/h. Pure minaprine (400 mg) in methanol (400 mL) in chapped glass bottle was directly irradiated with 5, 10, 20, 30, and 40 kGy dose. The methanolic solution of irradiated samples was immediately evaporated to remove the solvent and dried. The dried minaprine irradiated at 30 kGy dose showed the most improved NO production inhibitory effects in LPS-induced RAW264.7 cells.

### Isolation and quantitation of newly generated product

The irradiated minaprine reactant (265.6 mg) at 30 kGy dose was directly subjected to column chromatography over a YMC GEL ODS AQ 120-50S column (1.0 cm i.d. × 43 cm, particle size 50 μm) with 25% MeOH in H_2_O, to afford pure compound **2** (206.1 mg, *t*_R_ 6.3 min) (Fig. S1). HPLC analysis was conducted on a YMC-Pack ODS A-302 column (4.6 mm i.d. × 150 mm; 5 μm particle size; YMC Co., Kyoto, Japan), and the gradient solvent system initiated with 0.1% HCOOH (flow rate: 1.0 mL/min; detection: UV 280 nm; temperature: 40 °C), increased to CH_3_CN over 15 min. In addition, the purity of each individual compound isolated was also determined by HPLC with values more than 99%. The newly modified product (**2**) from minaprine (**1**) was monitored using their retention times (*t*_R_) and compared with original mianprine. Stock solutions of minaprine (**1**) and minaprinol (**2**) were prepared in MeOH, each at 1 mg/mL. Working solutions were then obtained, as mixtures of these stock solutions after serial dilutions with methanol, to achieve five concentration levels in the range of 1 to 0.0625 mg/mL. The working solutions were filtered through a syringe filter (Fisher Scientific, Fair Lawn, MJ) prior HPLC injection. After this, the linearity was determined by linear regression analysis of the integrated peak areas (*Y*) vs. the concentration of each standard (*X* mg/mL) at five different concentrations.

Minaprinol (**2**): Yellowish oil. UV λ_max_ MeOH (log ε): 219 (1.65), 284 (3.90) nm. ^1^H- and ^13^C-NMR, see Table 1. ESIMS *m/z* 331 [M + H]^+^, HRESIMS *m/z* 331.2126 [M + H]^+^ (Calcd for C_18_H_27_N_4_O_2_, 331.2128) (Fig. S2-S9).

### Cell culture

The RAW 264.7 cell line (mouse) was obtained from the Korean Cell Line Back (Seoul National University, Seoul, Korea) and DH82 cell line (canine) was obtained from the American Type Culture Collection (ATCC, Rockville, MD, USA). The macrophage cells were cultured under sterile conditions at 37 °C in a humidified atmosphere containing 5% CO_2_. The macrophage cells culture medium consisted of Dulbecco’s modified eagle medium (DMEM, GIBCO Invitrogen Corp., Carlsbad, CA, USA) of supplemented with 10% fetal bovine serum (FBS, GIBCO Invitrogen Corp.,), and 1% penicillin/streptomycin (GIBCO Invitrogen Corp).

### Cell viability assay

The effects of the newly formed product from irradiated minaprine on cell viability in RAW 264.7 and DH82 cells was evaluated by 3-(4,5-dimethylthiazol-2-yl)-2,5-diphenyltetrazolium bromide (MTT) method^26^. Cells were seeded at a density of 5 × 10^4^ cells/well into 96-well plates and incubated for 24 h at 37 °C. The cells were treated with minaprine and isolated compound **2** (50, 100, and 200 μM) in dissolved free medium, and incubated for 24 h at 37 °C. A solution of MTT (0.5 mg/mL) was added to each well and the plates were incubated for 3 h at 37 °C to allow the reaction to take place before removal of the culture medium, then the produced formazan blue was dissolved in dimethyl sulfoxide (DMSO). Cell viability was determined using a spectrophotometer and the absorbance was measured at 570 nm. The control group was considered 100%.

### Measurement of pro-inflammatory mediators and cytokine productions

The RAW 264.7 and DH82 cells were plated in a 96-well plate at a density of 5 × 10^4^ cells/well and incubated for 24 h at 37 °C. The cells were pre-treated with various concentrations (50, 100, and 200 μM) of isolated compound **2** for 2 h before incubating with LPS (0.1 or 1.0 μg/mL) for 24 h at 37 °C. Nitric oxide production was determined by the reaction of a macrophages culture supernatant with a Griess reagent^27^. The culture supernatant (100 μL) was mixed with the Griess reagent (100 μL) at room temperature and shaken gently for 20 min. Finally, a microplate reader was used to measure the absorbance of the reactants at 548 nm. The levels of PGE_2_ and cytokines were determined by ELISA using commercial reagent kits (BD Biosciences) according to the manufacturer's instructions.

### Western blot analysis

Sample treated RAW 264.7 and DH82 macrophage cells were harvested, lysed with radioimmunoprecipitation assay buffer (RIPA; Rockland Immunochemicals, Inc., Limerick, PA, USA). Cell debris was removed by centrifugation and the protein concentration was determined by bicinchoninic acid protein assay kit (Thermo Fisher Scientific, Inc.) according to the manufacturer's instructions. Cell lysates containing an equal amount of protein (30 μg) were prepared and separated by 10% SDS‑PAGE and 10 μg of cytosolic fractions and 15 μg of both mitochondria and debris fractions were loaded onto 12% SDS‑PAGE and transferred to polyvinylidene difluoride membranes (PVDF; Merck Millipore). The membranes were then blocked with 5% non‑fat milk in Tris‑buffered saline containing Tween‑20 (TBST) for 1 h at RT. The membranes were subsequently probed overnight at 4 °C with primary antibodies at dilution of 1:1,000 for anti-COX-2 (cat. #4842), anti-iNOS (cat. #2977), and anti‑GAPDH (cat. no. 2118) (all primary antibodies; Cell Signaling Technology, Danvers, MA, USA). For canine macrophages, the primary antibodies at dilution of 1:1000 for anti-COX-2 (cat. PA1-9032), anti-iNOS (cat. PA1-036), and anti-GAPDH (cat. PA1-987) were purchased from Invitrogen (Carlsbad, CA, USA). The blots were then incubated with secondary antibody horseradish peroxidase (HRP)‑conjugated anti‑rabbit IgG (cat. #7074 at 1:3000) both from Cell Signaling Technology (Danvers, MA, USA) for 2 h at RT. The membranes were incubated with the chemiluminescence (ECL) reagent (Thermo Fisher Scientific, Inc.) for protein band detection (Fig. S10).

### Quantitative reverse transcriptase polymerase chain reaction

Total RNA was isolated with the Qiagen RNeasy Plus Mini Kit according to the manufacturer’s protocol. Labo Pass™ cDNA synthesis Kit (Cosmogenetech) was used to synthesize cDNA from 1 μg total RNA per sample. The target cDNA was amplified using the following primers: iNOS forward: 5′-CCTGGTACGGGCATTGCT-3′,reverse: 5′-GCT CAT GCG GCC TCC TTT-3′, Arg-1 Forward: 5′-GGAATCTGCATGGGCAACCTGTGT-3′, reverse: 5’-AGGGTCTACGTCTCGCAAGCC A-3′, and GAPDH forward: 5′-TGGCAAAGTGGAGATTGTTGCC-3′, reverse: 5′-AAGATGGTGATGGGCTTCCCG-3′. The cDNA was used to evaluate the level of gene expression by qPCR using SYBR Green Master Mix (Bio-rad). The reaction mixture was incubated at 95 °C for 1 min, followed by 30 cycles of 95 °C for 10 s, 57 °C for 1 min and the relative iNOS and Arg-1 expression was calculated, respectively.

### Statistical analysis

All data were evaluated by the Student’s two-tailed *t*-test and results were considered statistically significant when the *p < 0.05 and **p < 0.01. Experiments were performed at least of three times independently.

## Supplementary Information

Supplementary Figures.

## Data Availability

The data underlying this article are available in the article and in its online supplementary material.
