# Supplementary material for: Novel aminopyridazine derivative of minaprine modified by radiolysis presents potent anti-inflammatory effects in LPS-stimulated RAW 264.7 and DH82 macrophage cells
Source: Sci Rep. 2023 Jul 5;13:10887. doi: 10.1038/s41598-023-37812-8 (PMC10322889; doi:10.1038/s41598-023-37812-8)
Supplement: Supplementary file 1 — Supplementary Figures. [file 41598_2023_37812_MOESM1_ESM.docx]

**Novel aminopyridazine derivative of minaprine modified by radiolysis presents potent anti-inflammatory effects in LPS-stimulated RAW 264.7 and DH82 macrophage cells**

**Gyeong Han Jeong ^a,b,#^, Hanui Lee ^a,b,#^, So-Yeun Woo ^a^, Hong-Ki Lee ^b^, Byung Yeoup Chung ^a^, Hyoung-Woo Bai ^a,b,c,*^**

^a^ Research division for Biotechnology, Advanced Radiation Technology Institute (ARTI), Korea Atomic Energy Research Institute (KAERI), Jeongeup 56212, Republic of Korea

^b^ Center for companion animal new drug development, Korea Institute of Toxicology (KIT), Jeongeup 56212, Republic of Korea

^c^ Radiation Biotechnology and Applied Radioisotope Science, University of Science and Technology (UST), Daejeon 34113, Republic of Korea.

* Corresponding Author. E-mail address: hbai@kaeri.re.kr (H.W.Bai)

^#^ These authors contributed equally to this work.

**Contents**

**Figure S1.** HPLC chromatograms of irradiated mianprine and the isolated compound **2**.

**Figure S2.** ^1^H NMR spectrum of compound **2** in CD_3_OD.

**Figure S3.** ^13^C NMR spectrum of compound **2** in CD_3_OD.

**Figure S4.** ^1^H-^1^H COSY spectrum of compound **2** in CD_3_OD.

**Figure S5.** HSQC spectrum of compound **2** in CD_3_OD.

**Figure S6.** HMBC spectrum of compound **2** in CD_3_OD.

**Figure S7.** NOESY spectrum of compound **2** in CD_3_OD.

**Figure S8.** HRESIMS spectrum of compound **2**.

**Figure S9**. Chemical structures of minaprine and isolated compound **2**.

**Figure S10**. Western blotting data for COX-2, iNOS, and GAPDH of compounds **1** and **2** in LPS-stimulated RAW264.7 and DH82 cells.

**
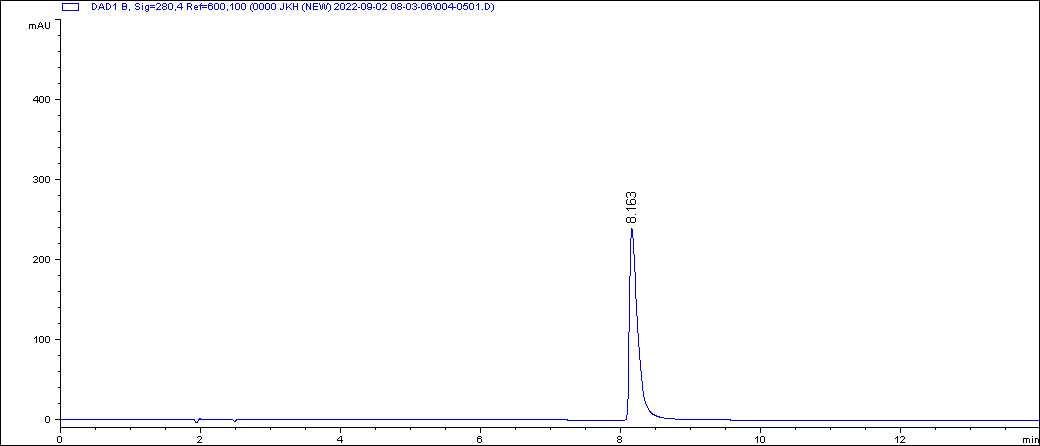

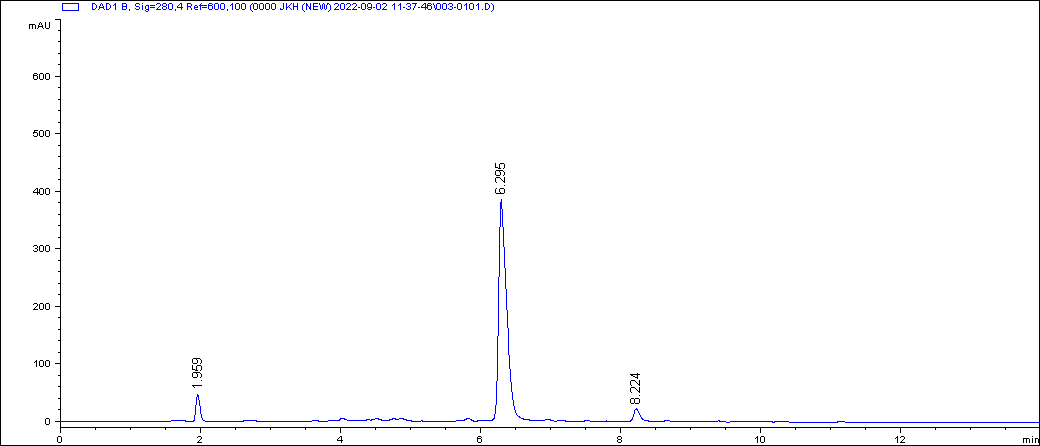

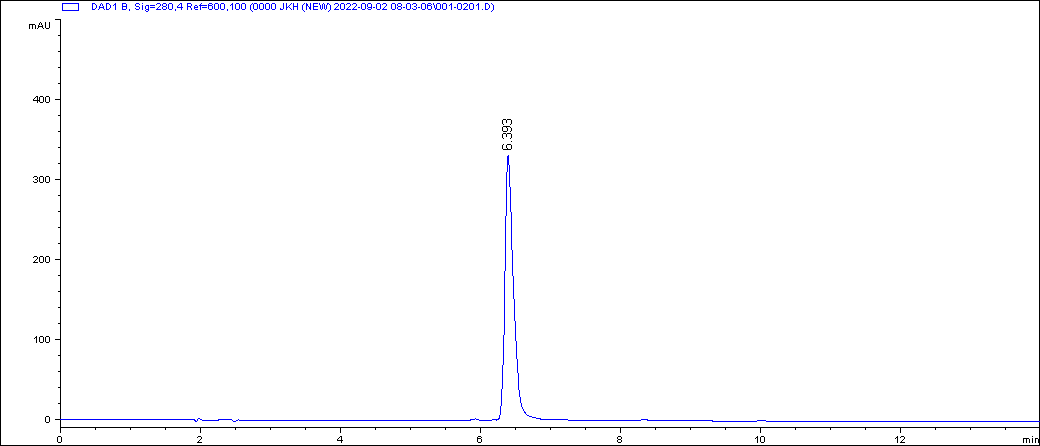
**

Compound **2**

Irradiated reactant at 30 kGy

Minaprine (**1**)

**Figure S1.** HPLC chromatograms of irradiated mianprine and the isolated compound **2**.


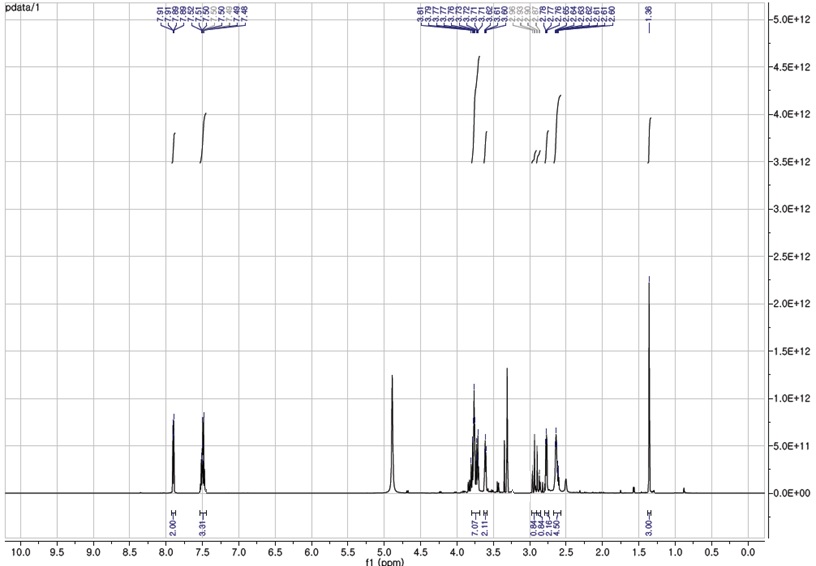


**Figure S2.** ^1^H NMR spectrum of compound **2** in CD_3_OD.


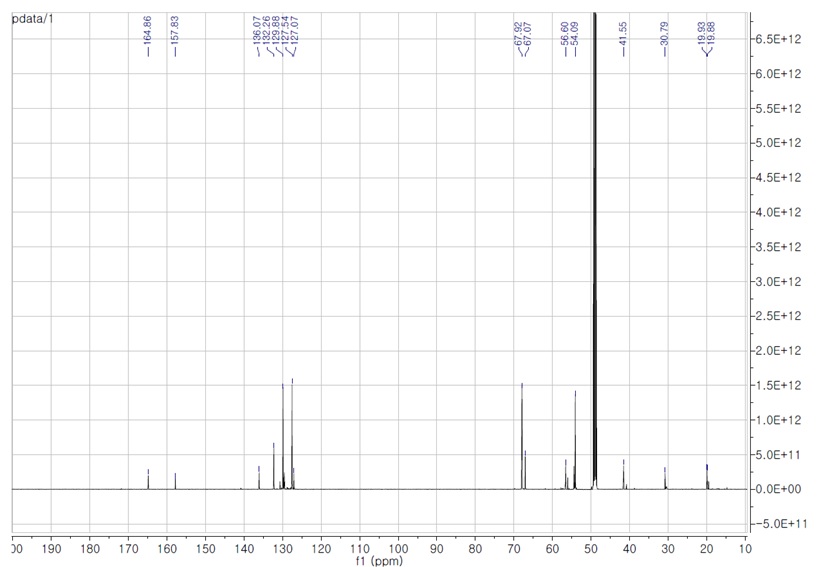


**Figure S3.** ^13^C NMR spectrum of compound **2** in CD_3_OD.


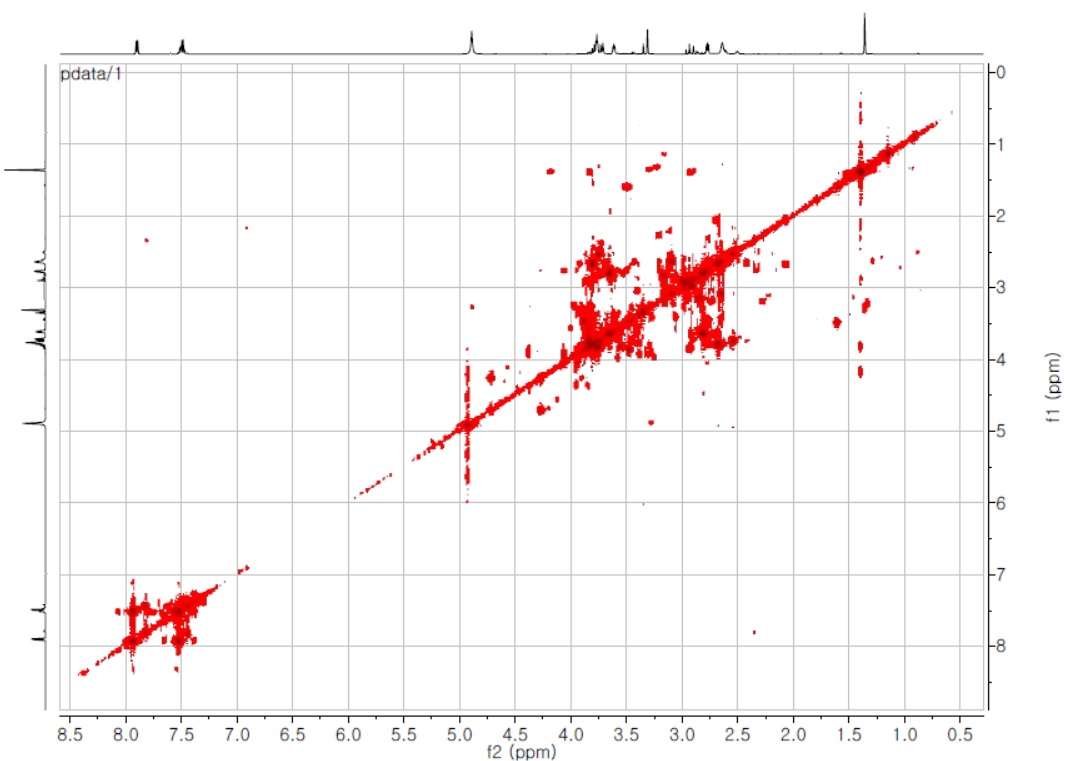


**Figure S4.** ^1^H-^1^H COSY spectrum of compound **2** in CD_3_OD.


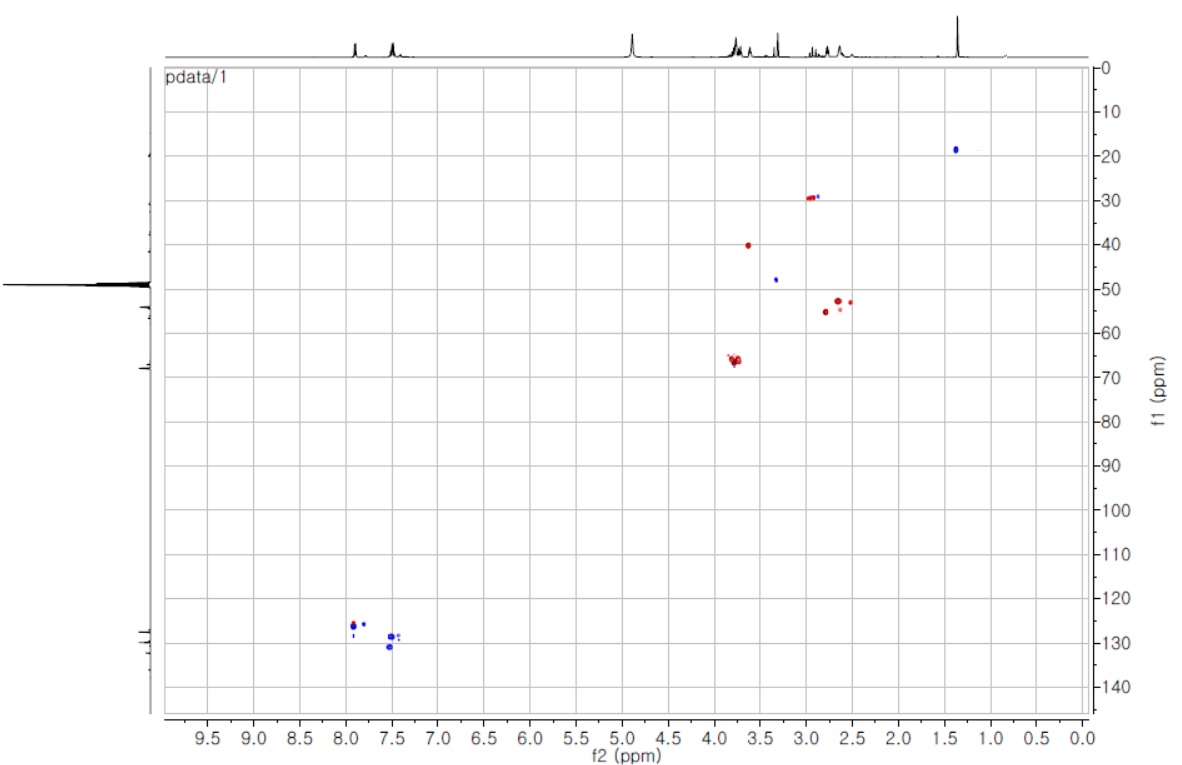


**Figure S5.** HSQC spectrum of compound **2** in CD_3_OD.


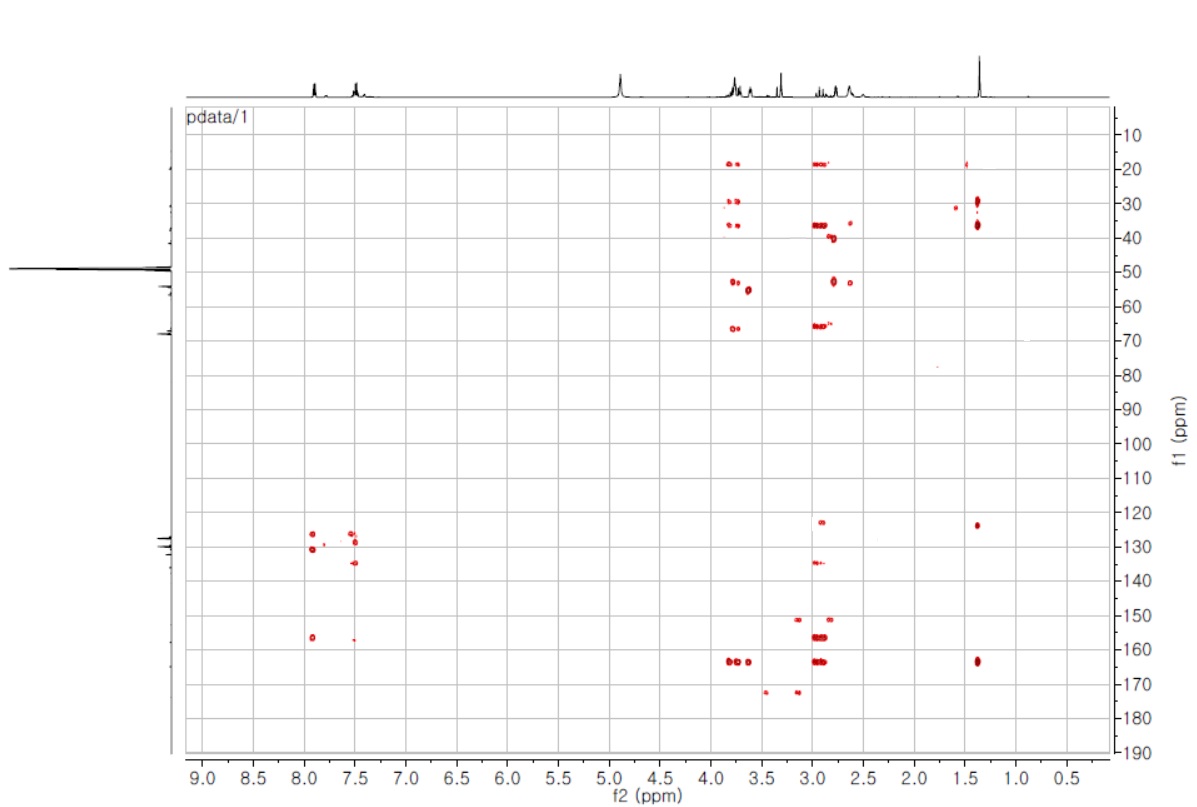


**Figure S6.** HMBC spectrum of compound **2** in CD_3_OD.


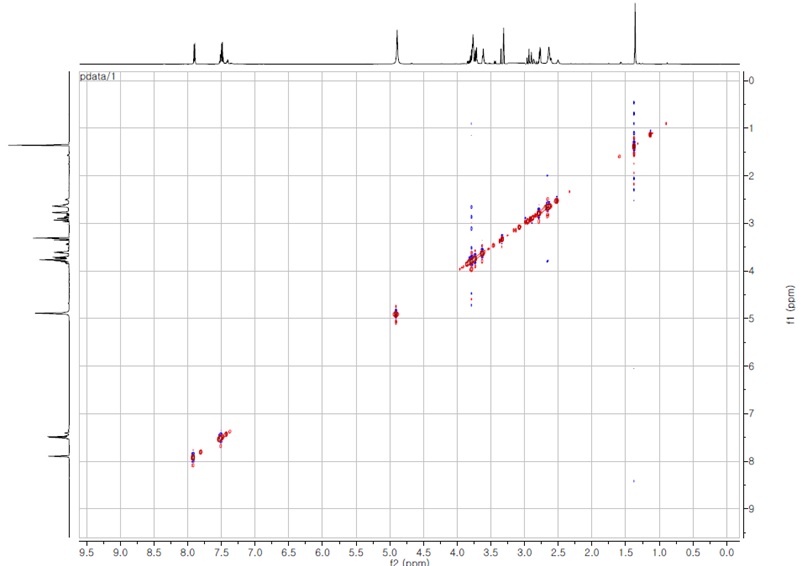


**Figure S7.** NOESY spectrum of compound **2** in CD_3_OD.

**
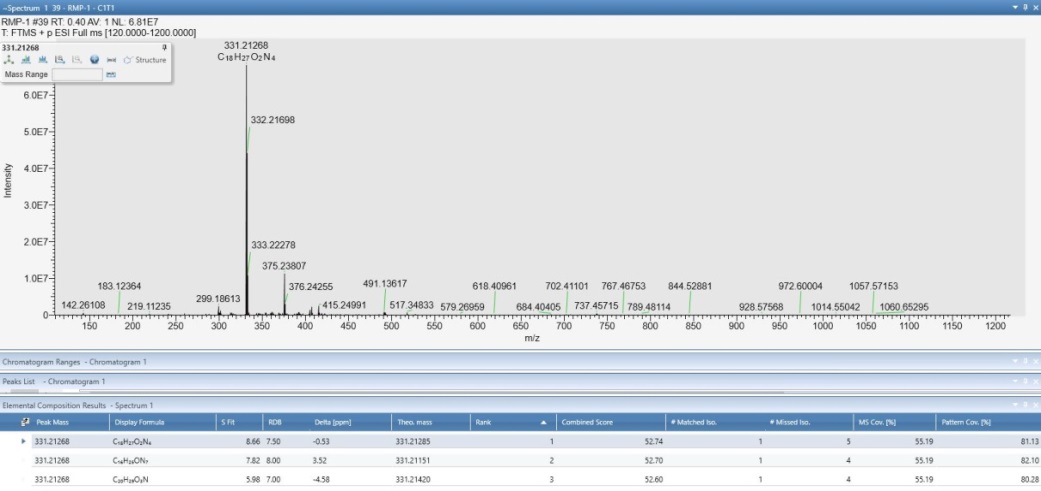
**

**Figure S8.** HRESIMS spectrum of compound **2**.

**Figure S9**. Chemical structures of minaprine and isolated compound **2**.

**
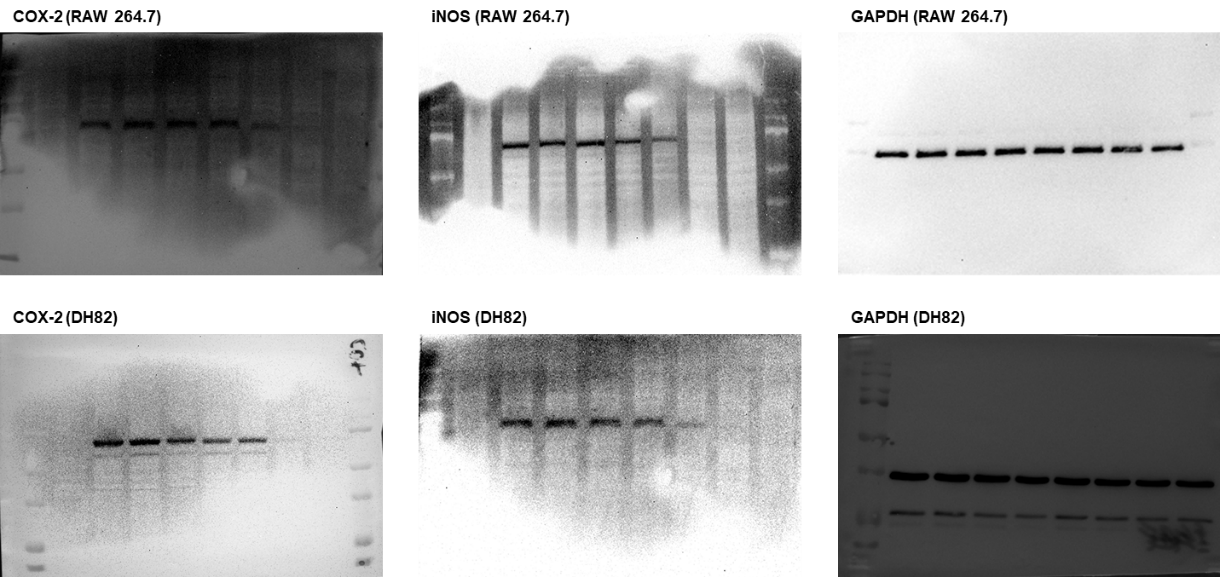
**

**Figure S10**. Western blotting data for COX-2, iNOS, and GAPDH of compounds **1** and **2** in LPS-stimulated RAW264.7 and DH82 cells.
